# Registration Law in Pennsylvania

**Published:** 1851-12

**Authors:** 


REGISTRATION LAW IN KENTUCKY.

We republish from the *Transactions of the Medical Society of the State of Pennsylvania*, the Pennsylvania law on the subject of registration. It is eminently adapted to the wants of this State, and we hope the Legislature of Kentucky will enact a law, with some modifications, to suit Kentucky customs, similar to the one we republish. We hope the physicians of the State are doing their duty towards this great cause.

*Bill Relative to the Registration of Marriages, Births, and Deaths, as passed by the Legislature of Pennsylvania—Session* 1850–51.

**Bill No. 649.—House of Representatives File. Read March 13th, 1851.**

*Whereas,* From the death of witnesses, and from other causes, it has often been found difficult to prove the marriage, birth, or death of persons, whereby the rignts of many have been sacrificed, and great wrongs have been done.

*And whereas,* Important truths deeply affecting the physical welfare of mankind, are to be drawn from the number of marriages, births, or deaths, that during a term of years may be contracted, or may occur within the limits of an extensive Commonwealth.

Therefore :—

Sec. 1. *Be it enacted by the Senate and House of Representatives of the Commonwealth of Pennsylaania in General Assembly met, and it is hereby enacted by the authority of the same,* That from and after the first day of July next, each register of wills in the several counties counties of this State, shall be supplied with separate books, in which he shall register, in the manner hereinafter directed, the marriages which may have been contracted, and the births and deaths which may have occurred within his county.

Sec. 2. That it shall be the duty of every clergyman, alderman, justice of the peace, clerk or keeper of the records of the religious society of Friends, and of every other person or society, by or before whom any marriage may hereafter be solemnized or contracted, to make at once a record of the same in a book to be kept for the purpose, and within the space of thirty days after such marriage, to return the same in the form of a certificate, duly signed by the person so certifying to the register of the county in which such marriage shall have been solemnized or contracted, whlch said record and certificate shall set forth, as far as the same can be ascertained, the full name of the husband, his occupation, and the name of his place of birth, and residence, the full name of the wife previously to the said marriage, the names of the parents of said husband, and of the parents of said wife, also the color of the parties, and the time, place where, and ceremony by which such marriage was contracted, and if pronounced by any clergyman or other person as aforesaid, the place of residence of such person.

Sec. 3. It shall be the duty of every physician, under whose care a birth shall hereafter take place, to make at once a record of such birth in a book to be kept for the purpose, and to return the same duly signed by him or her in the form of a certificate, to the register of the county in which such birth shall have taken place, in the manner, and within the period directed by the second section of this act, which said record and certificate shall set forth, as far as the same can be ascertained, the full name of such child, if any name should have been conferred, its sex, color, names of any other child or children living, the full name as well as occupation of its parent or parents; and for better identification, the full name previously to marriage of the mother of such child, the day, hour, and place in, and at which such birth occurred, and any circumstances connected with such birth that may be deemed of interest. In case snch birth shall have occurred without the superintendence of any person, and should no physician or other person be in attendance upon the parent immediately thereafter, it shall then become the duty of the parent or parents of such child, to return the same to the register in the manner, form, and within the period above required.

Sec. 4. Every physician, or surgeon, who shall hereafter be in attendance at the period of the death of any individual dying within this State, shall make at once a record of such death in a book to be kept by him for the purpose, and shall return the same duly signed in the form of a certificate, to the register of the county in which such individual may have resided at the time of death, in the manner and within the period directed in the second section of this act, which record and certificate shall set forth, as far as the same can be ascertained, the full name, sex, and color of the person deceased, and his or her age, also the name of his or her parents, the occupation, place of birth, the period, place, and disease, or cause of death, and within his knowledge the name of the burial ground in which interred, and if married at the time of death, the name of the husband or wife, as the case may be. And no person having the charge as sexton, or otherwise, of any vault or burying ground, within the City and County of Philadelphia, shall inter, or permit or cause to be interred, the dead body of any person in such vault or burying ground, without first procuring a copy of such record duly certified as aforesaid by the person who made it; which certificate as aforesaid when obtained he shall sign, and before the expiration of thirty days from the time at which he may have received it, shall return the same to the register aforesaid. And every physician, coroner, or other person, who shall neglect or refuse to furnish such sexton, or other person, previous to such interment within the said city or county, the certificate required by this section, and every sexton or other person having the care or superintendence of any vault or burying ground, who shall neglect to procure such certificate as aforesaid, or having procured it, shall neglect or refuse to return it as aforesaid, shall each for every such neglect or refusal, forfeit and pay the sum of five dollars, which sum shall be recoverable as debts of the like amount are recoverable by any person who may sue for the same. All certificates or returns required by this act, shall be signed by the parties so certifying.

Sec. 5. This act shall not be construed to prevent the registry of any marriage contracted, or birth or death happening, previously to its passage, within the limits of this State; nor of the marriage, birth, or death, of any person or persons who may have married, been born, or may have died elsewhere, but who were the child or children of the citizens of this State; nor of any marriage contracted previously or subsequently to the passage of this act, in any other part of these United States or their territories, or beyond the limits of the same: *Provided*, Either of the parties married were permanently residing in this State at the time, or at some time previously or subsequently to such marriage: such registrations shall be in the form already prescribed, and shall be kept apart from the current registrations, and in separate books, as is already required in the case of other registrations, but shall be embraced in the general index. The proof of every such marriage shall be as follows, to wit: in the case of a marriage by the person who pronounced it, or if such proof cannot be made, or the marriage shall have been contracted according to the manner of the religious society of Friends, then it shall be made by some one who was present thereat: in the case of a birth, by some one who has actual knowledge of the period at which such person was born, or of his or her parentage; and in the case of a death, by some one who actually saw such person dead, or who has actual knowledge of the fact. Such proof shall be under oath or affirmation, and shall be in its character satisfactory to the register: *Provided further*, that should any person feel aggrieved at the decision of the register aforesaid, he or she, or as the case may be, the next friend, or the representative of the person on whose behalf such application is made, shall have the right of an appeal to the Orphans' Court of the proper county, in which case the testimony so taken shall be sent up with the appeal to the said court, by the said register, accompanied by a written statement of his reasons for rejecting it as insufficient.

Sec. 6. That no letters of administration or letters testamentary shall be granted by any register upon the estate or effects of any person, hereafter dying within this State, or if granted, shall be valid, until the death of such person shall be duly certified to the said register, in order that the same may be duly registered according to the forms and provisions of this act, or as strictly in compliance therewith, as it may be in the power of the party so to do.

Sec. 7. That no appointment of any guardian to the person or estate to any minor, hereafter born, by any orphans' court within this State, shall be valid, until the date of the birth of such minor, and the date of the death, as well as the name of his or her parent or parents shall be duly registered according to the provisions of this act, or as strictly as the same can be complied with, unless from the death of any witness, or from some other cause deemed sufficient, upon strict investigation by the said court, such proof cannot be made, or cannot at the time be conveniently made, in which latter case it shall be made as soon as it may be practicable.

Sec. 8. That the said books or registers, or a certificate, duly certified by the register and authenticated by his seal of office, as containing a full copy of the record of any marriage, birth, or death, shall hereafter be admitted in any court of this State as *prima facie* proof of any marriage, birth or death: *Provided, however*, That should no such registration exist, the proof of any marriage, birth or death shall be sufficient, if made in the manner now or hereafter required under the decisions of the Supreme Court of this Commonwealth.

Sec. 9. That if any register shall not, within fourteen days after an application made to him to register any marriage, birth, or death, as aforesaid, register in the proper form and book, any such marriage, birth, or death, as the same shall be certified to him, unless he has sufficient cause, he shall forfeit and pay the sum of ten dollars, to be recovered as debts of the same amount are recoverable by any person who may sue for the same; the person aggrieved shall have the right of appeal provided by the 6th section of this act, and all certificates of marriages, births, or deaths, duly returned, shall be preserved and filed by the register.

Sec. 10. That if any person shall wilfully, knowingly, and falsely, swear or affirm to, or return any such certificate of such marriage, birth, or death, or if any register shall, wilfully and knowingly, make, or cause to be made in the said books, a false entry of such marriage, birth, or death, the said person or register so offending shall be punished by fine or imprisonment, or both, at the discretion of the Court having cognizance of such offences, the fine not to exceed eight hundred dollars, and the imprisonment not to exceed seven years.

Sec. 11. The registry of marriages, births, and deaths, shall be kept in separate books. There shall be general indexes to the records of all marriages, births, and deaths; which indexes of marriages, births, and deaths, respectively, shall be kept in separate books Opposite to each name in the said index shall be affixed not only the reference to the record, but the day of the month and year in which any marriage may have been contracted, or birth or death may have occurred, as the case may be.

Sec. 12. In order to secure uniformity, precision, and greater despatch, in the aforesaid registration, the said books shall contain (upon each page of the same, and along the margin at the side of the page) printed titles, duly numbered, opposite to which shall be left blanks or spaces in which entries shall be made corresponding to the particular subject of each of the said titles. The said titles in the books for registering marriages, shall be printed and arranged in the following words and order, to wit:—

*For Registering Marriages.*

1. Full name of the husband.
2. Name of the father of said husband.
3. Name of the mother of said husband.
4. Occupation of husband.
5. Residence of husband.
6. Birth-place of husband.
7. Full name of the wife, previously to marriage.
8. Name of the father of said wife.
9. Name of the mother of said wife.
10. The time when the marriage was contracted.
11. The place, town or township, and county where the marriage was contracted.
12. The color.
13. By what ceremony contracted.
14. Name of person pronouncing marriage.
15. Residence of person last named
16. Name of person signing the certificate.
17. Date of certificate.
18. Date of Registration.

*The titles in the books for Registering Births in the following words and order, to wit:—*

1. Full name of child.
2. Sex.
3. Color.
4. Names of other issue living.
5. Full name of father.
6. Occupation of father.
7. Name of the mother previously to marriage.
8. Hour, day of week, of month, and the year of birth.
9. Place, town or township, and county in which born.
10. Name of physician or other person signing the certificate, or on whose application registry is made.
11. Residence of such person.
12. Date of certificate.
13. Date of Registration.
14. Any additional circumstances.

*The titles in the books for Registering Deaths in the following words and order, to wit:—*

1. Full name of deceased.
2. Color.
3. Sex,
4. Age.
5. Name of father of deceased.
6. Name of mother of deceased.
7. Occupation.
8. Place of birth.
9. Name of wife of deceased.
10. Name of husband of deceased.
11. Date of birth and date of death.
12. Cause of death.
13. Name of the place, town or township, and county in which the person died.
14. Name and location of burial ground in which interred.
15. Name of person returning certificate.
16. Residence of such person.
17. Date of certificate.
18. Date of registration.

At the bottom of each page in each of said books, there shall be left a blank for the registry of any fact, which any act or acts of assembly hereafter enacted may require shall be registered, and all interlineations or erasures shall at the time be noted by the register.

Sec. 13. The said register, with the exceptions hereinafter named, shall receive, provided he comply with the provisions of this act, for registering any marriage, birth, or death, the sum of ten cents, which shall be paid to him, on his demand, by the treasurer of the proper county, except for registering any marriage contracted, or birth or death happening previously to the year one thousand eight hundred and fifty-one, in which case he shall receive twenty-five cents, to be paid by the party for examining witness, in which the testimony is reduced to writing and at length, twenty-five cents; for sending up the record upon an appeal, fifty cents; for granting a certified copy of the full record of any marriage, birth, or death, fifty cents; which shall also be paid to him by the party. He shall make no charges for administering an oath or affirmation, nor demand a fee from any person so registering. The Register for the City and County of Philadelphia shall receive but six cents for making any registry, as aforesaid, which shall be paid by the county treasurer as aforesaid.

All emoluments arising from the registration, except the amount paid by the several counties to the registers, which shall exceed the sum of one thousand dollars, shall be paid into the State Treasury. In order the more effectually to guard against injury from the loss by fire or otherwise, of the records of any county, and for other purposes, it shall be the duty of each register (under the penalty of twenty dollars for every neglect, recoverable as aforesaid), in each of the counties semi-annually, between the fifteenth and twentieth day of January and July in each year, to transmit a copy of the record of marriages, births, and deaths, before specified, with a copy of the index, duly certified by him to the Secretary of the Commonwealth, who shall file the same in his office, and annually transmit to the Legislature an abstract of the number of marriages, births, and deaths, which have occurred in each county in the State during each year next preceding the first day of January, and for which purpose the said Secretary shall furnish, at the cost of the Commonwealth, each of the said registers with proper blanks, prepared in the form required by the thirteenth section of this act; and shall also furnish to each of the said registers, at the cost of the Commonwealth, the blank books and indexes required by the first or any other section of this act; and shall also, at the same time, transmit a printed copy of this act, which shall be bound with said blank books, and such instructions on the subject of the law as he may see fit to prepare, and generally to do whatever may be required to carry into effect the provisions of this act.

Sec. 14. Every clergyman, justice of the peace, physician, sexton, or undertaker, whose duty it is to make any return under this act, shall, on or before the first day of October next, return his name and residence to the register of the county in which he may reside, who shall record the said return in a book to be kept for the purpose, to be provided at the expense of the county; and such person shall notify the register of his removal to any other place in or out of the said county, within thirty days after such removal, except where he may cease to act in a pro-essional or official capacity.

Sec. 15. All acts or parts of acts inconsistent herewith, or supplied as aforesaid, are hereby repealed.

